# Development of a Genome-Wide Oligonucleotide Microarray Platform for Detection of DNA Copy Number Aberrations in Feline Cancers

**DOI:** 10.3390/vetsci7030088

**Published:** 2020-07-07

**Authors:** Rachael Thomas, Joan U Pontius, Luke B Borst, Matthew Breen

**Affiliations:** 1 Department of Molecular Biomedical Sciences, College of Veterinary Medicine, North Carolina State University, Raleigh, NC 27606, USA; matthew_breen@ncsu.edu; 2 Comparative Medicine Institute, North Carolina State University, Raleigh, NC 27606, USA; 3 Laboratory of Genomic Diversity, Basic Research Program, Science Applications International Corporation-Frederick, Inc., National Cancer Institute-Frederick, Frederick, MD 21702, USA; joanpontius@gmail.com; 4 Present address: JP Solutions, Ellicott City MD 21042, USA; 5 Department of Population Health and Pathobiology, College of Veterinary Medicine, North Carolina State University, Raleigh, NC 27606, USA; lbborst@ncsu.edu; 6 Center for Human Health and the Environment, North Carolina State University, Raleigh, NC 27607, USA; 7 Cancer Genetics Program, UNC Lineberger Comprehensive Cancer Center, Chapel Hill, NC 27514, USA

**Keywords:** feline, cat, cancer, genomics, chromosome, comparative genomic hybridization (CGH)

## Abstract

The utility of the domestic cat as a model system for biomedical studies was constrained for many years by the absence of a comprehensive feline reference genome sequence assembly. While such a resource now exists, the cat continues to lag behind the domestic dog in terms of integration into the ‘One Health’ era of molecular medicine. Stimulated by the advances being made within the evolving field of comparative cancer genomics, we developed a microarray platform that allows rapid and sensitive detection of DNA copy number aberrations in feline tumors using comparative genomic hybridization analysis. The microarray comprises 110,456 unique oligonucleotide probes anchored at mean intervals of 22.6 kb throughout the feline reference genome sequence assembly, providing ~350-fold higher resolution than was previously possible using this technique. We demonstrate the utility of this resource through genomic profiling of a feline injection-site sarcoma case, revealing a highly disrupted profile of DNA copy number imbalance involving several key cancer-associated genes including *KIT,* *TP53*, *PTEN, FAS* and *RB1*. These findings were supported by targeted fluorescence in-situ hybridization analysis, which identified major alterations in chromosome structure, including complex intrachromosomal reorganization events typical of those seen in aggressive soft-tissue sarcomas of other species. We then characterized a second mass that was identified at a nearby site in the same patient almost 12 months later. This mass demonstrated a remarkably conserved genomic profile consistent with a recurrence of the original tumor; however the detection of subtle differences reflected evolution of the tumor over time. These findings exemplify the diverse potential of this microarray platform to incorporate domestic cat cancers into comparative and translational research efforts in molecular oncology.

## 1. Introduction

The unique natural history of the domestic cat, coupled with its intensive veterinary surveillance and growing popularity, offers unrivaled opportunities to model key biological processes, particularly those that are seldom encountered outside human and feline medicine. A vast spectrum of naturally occurring genetic disorders and other heritable pathologies has been described in the domestic cat, of which more than 160 are recognized as potential models of a human counterpart. As many as one third of these are considered rare in other common model systems such as the domestic dog and mouse [1,2]. Despite these many attributes, the cat has yet to occupy a prominent role in comparative and translational biomedical studies, particularly for non-heritable disorders, having been constrained for some years by a significant deficit in the essential genomic resources. While a comprehensive 7x-coverage reference genome sequence assembly for the dog was released in 2005 [3], by 2007 the feline toolbox contained only a draft ~1.9X shotgun assembly, capturing just ~65% of the euchromatic sequence [4]. Despite its inherently fragmented nature, this resource offered a preliminary template for comparative biomedical studies and for evaluation of aberrant feline genomes, such as those associated with the development and progression of a malignant phenotype.

Numerous human cancer studies have identified recurrent genomic alterations that correlate with discrete diagnostic subtypes, therapeutic responses and/or disease outcomes, offering molecular means for clinically predictive subclassification. The same principles are also well established for the domestic dog [5,6]. Previously, we utilized the draft 1.9x feline sequence assembly to construct a low-resolution microarray platform for detection of recurrent DNA copy number aberrations (CNAs) in feline cancers using large insert genomic clones, enabling detection of broad regions of tumor-associated aneuploidy using comparative genomic hybridization (CGH) analysis [7]. The microarray revealed numerous recurrent CNAs among a cohort of 46 feline soft tissue sarcomas, indicative of extensive, non-random genomic instability. Of particular note, we identified subchromosomal regions whose copy number status differed significantly between two related tumor subtypes that share similar histopathology at diagnosis but which typically show contrasting clinical behavior and outcome. Our initial findings thus supported the potential for a molecular means to aid distinction between these tumor subtypes, which in turn has implications for defining prognosis and selection of appropriate treatment strategies. The low resolution of this first-generation array, however, limited the ability to determine the precise boundaries of these CNAs, and in turn to assess their gene content in the search for clinically predictive alterations.

With a more comprehensive feline reference genome assembly still in development, we embarked on an in-silico strategy that instead used a well-characterized dog microarray design to guide the construction of a second-generation feline CGH platform. Following the integration of our arrayed probe set into the most recent cat reference sequence assembly build [8], this platform now provides ~350-fold higher overall resolution than our first generation array [7], bringing feline cytogenomic resources more closely in line with those of other model systems. 

## 2. Materials and Methods

### 2.1. Design and Construction of the Feline Oligonucleotide CGH Array

In the absence of an assembled, anchored and annotated cat genome sequence at the time this study was initiated, a commercially available dog oligonucleotide array CGH (oaCGH) platform (design ID 025522, Agilent Technologies, Santa Clara, CA) was used as a template for in-silico design of a feline counterpart. This strategy was based on the assumption that the broadly uniform genomic distribution of array probes in this well-characterized dog design [9,10,11,12] would provide landmarks for directing the development of a feline platform with similar specifications, without prior need for a comprehensive cat reference sequence assembly. The canine array comprises 171,534 unique repeat-masked ~60–mer oligonucleotide probes distributed at mean intervals of ~13kb along each dog autosome and dog chromosome X (cfaX) [3]. The mean probe melting temperature (T_m_) was calculated as 80 ˚C (range 71–85 °C, median 80 °C) with a mean GC content of 36% (range 18–58%, median 37%). For development of the feline array, the nucleotide sequence of each unique dog probe was first aligned with the canFam3 dog genome assembly using BLAT [13]. This alignment was then extended by adding 100 nucleotides of matching canine sequence to each end of the original dog probe sequence. The resulting extended dog probe sequences (approximately 260 nucleotides in length) were used to search for the most highly conserved orthologous regions within the ~10,000 unplaced scaffolds comprising a preliminary 10x draft of the cat genome assembly (Wes Warren, pers. comm.), using megaBLAST [14]. Unambiguous canine–feline alignments were then evaluated using ClustalW [15] to extract the cat ortholog of the original dog ~60-mer probe sequence. By reference to the feline sequence data directly flanking this orthologous region, the length of each cat probe was then adjusted by addition or deletion of nucleotides to result in a T_m_ and GC content consistent with that of the original dog probe. Each of the candidate feline probe sequences was then mapped back to the preliminary 10x draft of the cat genome sequence assembly to confirm alignment with a unique location. The resulting probes were synthesized in a four-plex microarray format using Agilent SurePrint G3 technology, and used for DNA copy number profiling as described below. Upon the release of the felCat9 cat reference genome sequence assembly [8], each cat probe was remapped using BLAT [13] to assess their relative distribution and to interpret genomic profiling data in context with the architecture of the cat karyotype. 

### 2.2. Experimental Validation of the Feline oaCGH Array Design

The performance of the feline array design was investigated first by performing a sex-mismatch hybridization that assessed the ability to detect known DNA copy number imbalances within a normal balanced background. Female and male feline DNA pools, each representing constitutional DNA from ten clinically healthy individuals [7], were labeled with Cyanine3-dUTP and Cyanine5-dUTP, respectively, by random priming (SureTag Genomic DNA Enzymatic Labeling Kit, Agilent Technologies). The resulting pools of differentially-labeled female and male DNA were combined and hybridized onto the microarray as described previously [16] and the array was scanned at 3 µm resolution using an Agilent G2565CA scanner.

Scanned array image files were processed using Feature Extraction version 10.10 (Agilent Technologies) and imported into Nexus Copy Number version 10 (Biodiscovery, El Segundo, CA). Raw data were filtered to exclude probes exhibiting non-uniform hybridization or signal saturation. Recurrent CNAs within each tumor were defined using the FASST2 segmentation algorithm in Nexus Copy Number, based on a minimum window of three consecutive probes with log_2_ tumor: reference values ≥0.201 (copy number gain) or ≤−0.234 (copy number loss). High amplitude gains and losses within individual tumors were defined using default log_2_ tumor: reference values of ≥1.14 and ≤−1.1, respectively. Discrete genomic regions are reported herein according to their chromosomal location and Mb position in the felCat9 genome assembly [8]. Predicted genes and uncharacterized coding sequences located within regions of CNA were defined using the ‘RefSeq’ and ‘xenoRefSeq’ tracks of the felCat9 genome sequence browser hosted at http://genome.ucsc.edu/. 

### 2.3. DNA Copy Number Profiling of a Feline Injection-Site Sarcoma 

The cat oaCGH array design underwent further assessment through DNA copy number profiling of a feline injection-site-associated sarcoma (ISS) case, which had undergone prior molecular cytogenetic analysis at low resolution [7]. This tumor was selected due to its highly disrupted DNA copy number profile involving the majority of feline chromosomes, with marked variation in the amplitude of detected genomic imbalances. Briefly, this highly aggressive mass (referred to as ISS-19a) was surgically excised from the dorsal midline of the thoracic area of a 14 year old male domestic medium-hair cat, a site consistent with the site of FVRCP vaccination 13 months previously. A representative specimen was fixed in formalin and subjected to independent review by two board-certified veterinary pathologists [7]. After histologic examination of the excised mass indicated clean margins, the owners elected not to pursue ancillary treatment, and the patient was monitored regularly for signs of recurrence. A second rapidly growing mass (ISS-19b) was identified at a nearby site 11 months later. Histologic examination of formalin-fixed tissue from ISS-19b was suggestive of a recurrence of the original tumor. The owners elected not to pursue further treatment, and due to the poor outlook the patient was euthanized. Fresh tissue specimens from each mass were used for DNA isolation and for initiation of primary explant cultures, which were harvested for chromosome preparations at passage two, as described previously [7]. 

Tumor DNA from each mass (labelled with Cyanine3-dUTP) was evaluated independently by oaCGH analysis, as described above, using the pool of male constitutional DNA (labelled with Cyanine5-dUTP) as a common reference. Targeted multicolor fluorescence in-situ hybridization (FISH) analysis of metaphase and interphase chromosome preparations from both ISS-19a and ISS-19b was performed using a pool of differentially-labeled large insert genomic probes representing four key cancer-associated genes, as described previously [7] (BACPAC Resources, Children’s Hospital Oakland Research Institute, Oakland, CA). The same FISH probes were also hybridized to metaphase chromosome preparations generated from peripheral blood lymphocyte cultures from two unrelated, clinically healthy donor cats of mixed breed, to verify the expected chromosome location of fluorescent signals in normal individuals. The copy number and genomic distribution of each probe was recorded in 30 representative cells from each of the two ISS specimens and the controls.

## 3. Results

### 3.1. Guiding Development of a Feline oaCGH Array Design Using a Canine Template

Of the 171,534 unique probe sequences present in the existing canine oaCGH design, 58,255 (34%) did not identify a robust alignment with an orthologous region of the draft 10x cat genome assembly, and these regions were not pursued. Of the 113,279 successful alignments between dog probes and the cat sequence assembly, 1512 yielded an exact match, enabling the ~60-mer probe sequence to be incorporated directly into the new feline array design. A further 83,488 non-identical feline orthologs of dog probe sequences met the required T_m_ and GC content parameters, and were used directly in the feline array. For the remaining 28,279 probes, minor adjustment of the start and/or end position of the feline target sequence allowed these criteria to be met in 27,400 instances. The remaining 879 regions were excluded since the nucleotide composition of the available feline sequence precluded the identification of probes that met the required T_m_ and GC content parameters. 

**Figure 1:**
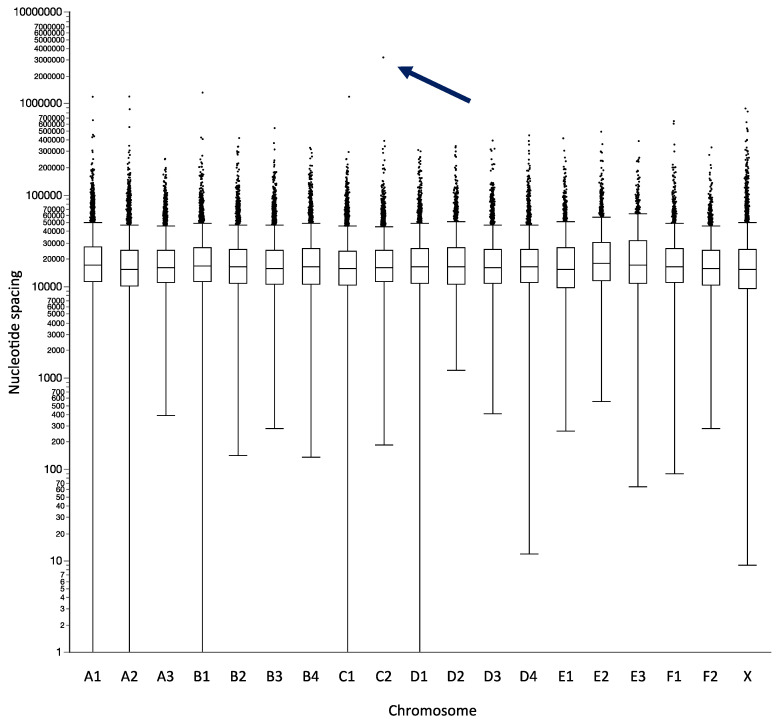
Outlier plot summarizing probe spacing on the feline oligonucleotide array comparative genomic hybridization (oaCGH) array. The x-axis shows each feline autosome and the X chromosome, and the y-axis shows the nucleotide intervals between consecutive probes on each chromosome, using a logarithmic scale. Each box delineates the interquartile range of interval sizes between probes, showing that 50% of all intervals lie within a close range of approximately 10kb to 30kb. The central horizontal bar within the interquartile range indicates the median probe spacing for that chromosome. This demonstrates the global uniformity of probe distribution, with median spacing ranging from 15.4kb (fcaE1) to 18.1kb (fcaE2), and an overall mean genome-wide spacing of 22.6kb. The vertical bars extending from each box define the minimum and maximum distances between two consecutive probes, excluding large ‘outlier intervals’, which are shown as individual datapoints. Outliers are defined as intervals that are larger than the 3rd quartile value + 1.5x the interquartile range. The ~3.2Mb outlier interval on fcaC2 (arrowed) resulted from loss of a cluster of probes mapping to a large scaffold in the preliminary felCat5 assembly that could not be integrated reliably into the more advanced felCat9 assembly.

The 112,400 candidate probes that progressed through to this phase were developed into the feline oaCGH array design used in the present study. Of these, 1944 subsequently produced inconclusive map assignments when anchored into the most recent feline reference sequence assembly build, felCat9 [8], and were excluded from downstream analysis. Thus, the final design comprises 110,456 probes with thermodynamically matched T_m_ and GC content and a unique and unambiguous physical assignment within the felCat9 reference genome sequence assembly. This provides a mean genome-wide distribution of one probe every 22.6 kb, distributed across the 2.5 Gb of sequence from 19 assembled cat chromosomes, yielding an average of 44 probes/Mb (Figure 1, Table 1). The absence of probes representing the cat Y chromosome (fcaY) reflects the female origin of both the cat and dog reference genome sequence assemblies. Overall, 94.0% of intervals between consecutive probes were smaller than 50kb, and 99.1% were smaller than 100kb. Twenty two intervals measured >1Mb in size, of which all but four represented highly repetitive centromeric regions that precluded the design of unique probes. Figure 1 shows a visual summary of probe distribution for each chromosome, and additional details are provided in Table 1.

### 3.2. Experimental Evaluation Using Sex-Mismatched Reference DNA 

**Figure 2:**
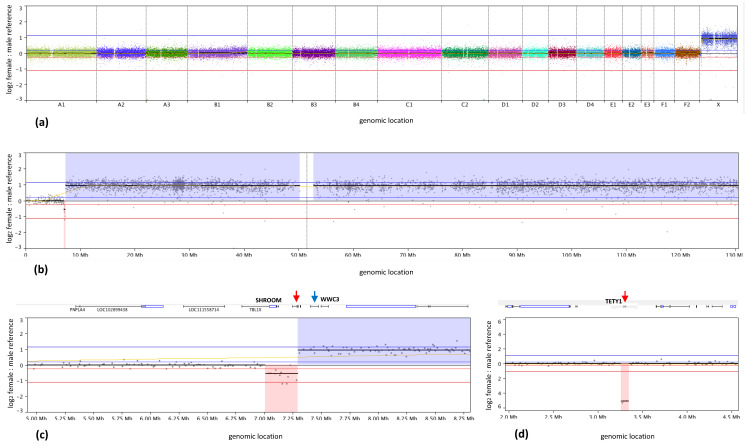
Genomic profile of female reference DNA hybridized against male reference DNA. Feline genomic locations are plotted along the x-axis, and relative log_2_ fluorescence intensity ratios are plotted on the y-axis. Each datapoint represents the female:male DNA copy number ratio (y-axis) of the corresponding arrayed probe sequence as determined by oaCGH analysis. The horizontal midline (y = 0) indicates relative genomic balance between the female and male reference DNA pools. Data points lying above the midline indicate a relative DNA copy number increase in the female reference pool, and those below the midline indicate a relative copy number decrease. The blue and red horizontal lines closest to y = 0 represent the thresholds for classification of relative DNA copy number gain and loss (log_2_ ratio ≥0.201 and ≤−0.234, respectively). The outer blue and red horizontal lines represent the thresholds for classification of high amplitude copy number gain and loss (log_2_ ratio ≥ 1.14 and ≤-1.1, respectively). Datapoints from each chromosome are shown in a different color to aid interpretation. The horizontal yellow line shows the moving average of relative copy number between the two DNA samples hybridized onto the array. **(a)** Balanced DNA copy number status was evident along all autosomes, with the exception of two regions, located on fcaA2ptel and fcaF2qmid (see main text for details). **(b)** Enlarged copy number profile for fcaX, demonstrating the expected relative copy number increase along the length of the chromosome in female versus male reference DNA (region shaded in blue), with the exception of a 7.3Mb region at fcaXptel. This region of balance represents the feline pseudoautosomal region (PAR) that is shared by the sex chromosomes. The central gap in probe distribution coincides with the highly repetitive centromeric region. **(c)** Enlarged view of the putative feline PAR boundary, indicating that the transition from the PAR to the unique fcaX sequence occurs between the *SHROOM* and *WWC3* genes. **(d)** Enlarged view of a 2.5Mb interval on fcaA3ptel, showing a 48kb region of relative copy number loss in female versus male reference DNA, containing the *TETY1* locus. This reflects the relative difference in gene dosage of this region in females versus males, due to the presence of multiple copies of a conserved sequence motif on fcaY.

Pairwise hybridization of differentially labeled male and female reference DNA pools exhibited the expected balanced copy number of each autosome, and relative copy number increase along fcaX in females versus males (Figure 2a). The mean log_2_ female:male signal intensity value for all probes along fcaX was 0.889 (expected theoretical value = 1), compared to 0.002 for autosomal regions (expected theoretical value = 0). Examination of relative copy number along fcaXp from sex mismatch hybridizations detected transition from balanced to unbalanced status within the interval fcaX: 6,976,314–7,309,991 bp (Figure 2b,c). The probes flanking the site of abrupt transition to relative genomic gain delineated the breakpoint of the feline pseudoautosomal region (PAR) within a 25.7kb window between the cat *SHROOM2* and *WWC3* genes, with the latter lying within the fcaX-specific region. When the putative feline PAR was excluded from sex-mismatch analysis, the mean log_2_ female:male value along fcaX increased to 0.919 (expected theoretical value = 1). These data provide strong support for the ability of the array design to detect relative DNA copy number imbalances with a high degree of sensitivity and accuracy.

Sex mismatch hybridizations identified an unexpected region of autosomal DNA copy number imbalance at fcaA3p:3,275,369–3,323,423 bp. Here, three consecutive probes, spanning 48 kb, showed a relative DNA copy number loss in the female reference compared to the male reference (Figure 2d). This interval contains the feline *TETY1* locus (*Felis catus* testis expressed transcript on Y–1 precursor). A second region of autosomal imbalance was detected at fcaF2:40,867,764–41,184,038 bp, represented by 18 probes spanning 316 kb and showing relative copy number loss in the female versus male reference. Aside from partial overlap with the *RUNX1T1* gene at the distal end of this region, no other genes are annotated within this interval. 

### 3.3. Cytogenomic Evaluation of Tumor Specimens 

**Figure 3:**
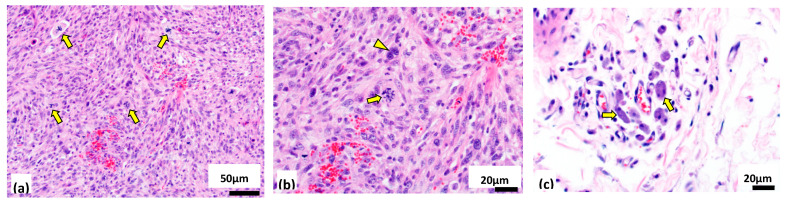
Histologic evaluation of representative hematoxylin and eosin--stained sections of tumor ISS-19a. **(a)** Assessment at low magnification (200x) demonstrated high mitotic activity (arrows) and an interwoven growth pattern composed of bundles and streams of fusiform to polygonal mesenchymal cells with moderate amounts of eosinophilic cytoplasm surrounding large, irregularly round nuclei. **(b)** Evaluation at higher magnification (400x) identified anaplastic cellular features, frequent anisocytosis and anisokaryosis, multinucleated giant cells (arrowhead) and bizarre mitoses (arrow). **(c)** At the periphery of the tumor was an inflammatory infiltrate composed predominantly of small lymphocytes and macrophages (arrows) bearing an intracytoplasmic blue-grey material, consistent with the presence of aluminum-based adjuvant. Histologic features were highly conserved between ISS-19a (56 mitotic figures per ten 400x fields of view) and ISS-19b (47 mitotic figures per ten 400x fields of view).

**Figure 4:**
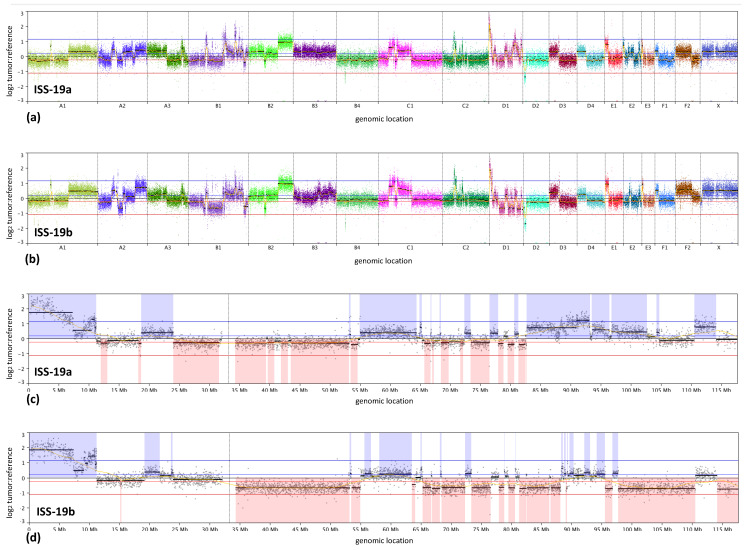
Genome-wide DNA copy number profiles derived from array-CGH analysis of ISS-19a and ISS-19b. (**a**) Genome-wide profile of the original tumor (ISS-19a) following resection. The tumor exhibits extensive copy number imbalance throughout the genome, evident as deviation from the horizontal midline, with only fcaB4 showing a grossly balanced copy number. (**b**) Genomic profile of a mass obtained from an area adjacent to the original tumor site 11 months after initial resection (ISS-19b). Comparison of these profiles demonstrates extensive conservation in their genome-wide copy number profiles, but reveals subtle differences both in the distribution and amplitude of DNA copy number aberrations (CNAs). Included among these were regions of higher amplitude deletion on fcaA2, fcaB1 and fcaB2 in ISS-19b that did not exceed the threshold for classification as copy number imbalance in the original tumor. (**c**) and (**d**) show enlarged data from the full length of fcaD1 in ISS-19a and ISS-19b, respectively. Intervals shaded in blue indicate DNA copy number gain in the tumor, and red shading indicates copy number loss. The vertical line at ~34Mb on the x-axis represents the centromere. Comparison of these profiles reinforces the vast, but highly conserved, genomic complexity exhibited by both tumors, consistent with marked chromosomal reorganization.

Figure 3 shows representative images of tumor histology for ISS-19a, which closely resembled that of ISS-19b. The microscopic appearance of both lesions was consistent with a diagnosis of aggressive fibrosarcoma, with the presence of adjuvant supporting an association with a prior injection event. Chromosome enumeration of primary cell cultures initiated from both masses showed modal chromosome numbers of 47 (range 46–50), indicative of moderate hyperdiploidy relative to the normal n = 38 for the domestic cat. oaCGH analysis identified extensive deviation from a balanced copy number status in both specimens, relative to the common reference pool of clinically healthy male constitutional DNA, reflecting a high degree of both structural and numerical instability (Figure 4a,b). Comparison of profiles from ISS-19a and ISS-19b demonstrated global conservation in the location of genomic imbalances, although the amplitude of these aberrations was broadly higher in the tumor reoccurrence (ISS-19b, Figure 4b), suggestive of increased clonality and/or reduced contamination by normal stromal cells. Among the aberrations evident in ISS-19a and ISS-19b were imbalances that spanned the length of a chromosome, including elevated copy number of fcaX in the male patient, compared to the clinically healthy male reference pool. More common was discordance in the relative copy number status of chromosome arms, suggestive of segmental aneuploidy resulting from centromeric fission and subsequent independent segregation (for example, gain of fcaD3p and 4p versus balance of fcaD3q and 4q). The majority of aberrations reflected discrete regions of copy number instability that varied in direction (gain/loss) and relative amplitude (degree of gain/loss) along the length of the chromosome. Notable among these was fcaD1, for which both ISS-19a and ISS-19b showed multiple distinct segmental changes in state (gain, loss or balance) along the length of the chromosome, suggestive of substantial intrachromosomal reorganization. The most striking of these was a high-level amplification event encompassing the first 11Mb of fcaD1ptel in both masses. The genomic profiles along fcaD1 were highly conserved between both masses, although ISS-19a showed copy number gain of fcaD1qdist, while this region was partially deleted in ISS-19b (Figure 4c,d). 

**Figure 5:**
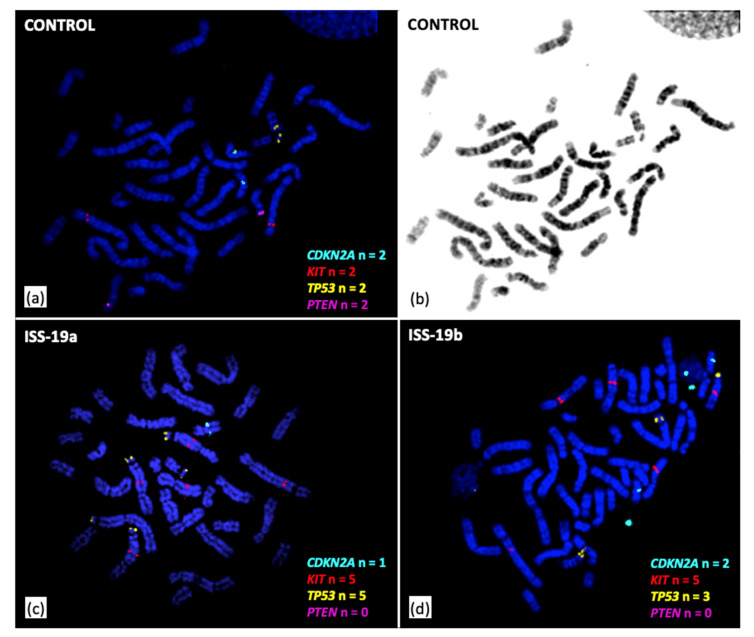
Targeted fluorescence in situ hybridization (FISH) analysis of ISS-19a and ISS-19b. **(a)** Differentially labeled FISH probes representing four genes were hybridized to control metaphase chromosome preparations from clinically healthy donor cats. This demonstrated the unique localization of these probes to fcaD4:46.13 Mb (*CDKN2A/B*, aqua probe), fcaB1:162.44 Mb (*KIT*, red probe), fcaE1:2.49 Mb (*TP53*, gold probe) and fcaD2:7.42 Mb (*PTEN,* purple probe), with the expected copy number (n = 2) in all control cells evaluated. **(b)** DAPI-banded image of the metaphase spread shown in (a), enabling confirmation of the expected chromosome location for each probe. **(c)** and (**d)** Representative images of FISH analysis of individual cells from ISS-19a and b, respectively, indicating the DNA copy number of the same panel of four probes. Both specimens exhibited hyperdiploidy and evidence of complex karyotypic reorganization. The copy number of *CDKN2A/B* was grossly balanced within the population of cells scored for both specimens (mean 1.7 in ISS-19a and 2.0 in ISS-19b). Both *KIT* and *TP53* showed elevated copy number, with a mean of 5.8 and 4.9, respectively, in ISS-19a, and 5.0 for both probes in ISS-19b. The majority of cells in both specimens exhibited one or more derivative chromosomes harboring both *KIT* and *TP53* signals (87% of ISS-19a cells and 94% of ISS-19b cells), suggestive of fusion between regions of fcaB1 and E1. *PTEN* probe signal was undetectable in both ISS-19a and b, indicative of homozygous deletion. Data from FISH analysis were consistent with those obtained by oaCGH analysis, and in combination support the origin of ISS-19b as a recurrence of the original ISS-19a mass.

These findings were examined further in conjunction with FISH analysis of selected genomic intervals harboring key cancer-associated genes. Detection of copy number increase of the distal 13.2 Mb of fcaE1p by oaCGH analysis identified gain of the *TP53* gene, located at fcaE1p:2.55 Mb. This was supported by FISH analysis, which showed a mean of 4.8 copies of *TP53* in cells from ISS-19a, and a mean of 5.0 copies in cells from ISS-19b (compared to the expected n = 2 status). In contrast, oaCGH analysis identified high amplitude aberrations in both masses that were consistent with homozygous deletion of the tumor suppressor genes *RB1* (fcaA1p:22.92 Mb), *PTEN* (fcaD2p:7.61 Mb), and the *FAS* cell surface death receptor gene (fcaD2p:8.65 Mb). In turn, while the *PTEN* FISH probe showed normal diploid status in control specimens, no *PTEN* FISH probe signal was detected in any cells evaluated for ISS-19a and ISS-19b, consistent with homozygous deletion (Figure 5a,b). 

oaCGH analysis demonstrated borderline deletion of the *CDKN2A* and *CDKN2B* tumor suppressor gene loci (fcaD4q:48.18 Mb) in ISS-19a and balanced copy number status of this region in ISS-19b. This was supported by FISH analysis, which showed a mean *CDKN2A/B* copy number of 1.7 and 2.0 within cells from ISS-19a and ISS-19b, respectively (Figure 5c,d). FISH analysis also revealed high-level amplification of the *KIT* oncogene (fcaB1q:164.0 Mb), with mean *KIT* gene copy numbers of 5.8 and 5.0 within cells from ISS-19a and ISS-19b, respectively, consistent with oaCGH analysis. Interestingly, while *KIT* and *TP53* are located on different chromosomes in the normal cat karyotype (fcaB1 and fcaE1, respectively), FISH analysis identified a derivative chromosome carrying both *KIT* and *TP53* probe signals in 87% of metaphases from ISS-19a and 94% from ISS-19b (Figure 5c,d). This indicated fusion of regions from fcaB1 and fcaE1 in the formation of an aberrant chromosome structure; however the extensive physical distance separating the *TP53* and *KIT* signals, and the comparatively large size of the derivative chromosome, was suggestive of a complex structural reorganization rather than a simple fusion event.

## 4. Discussion

We present a novel microarray platform that surveys the domestic cat genome for DNA copy number aberrations with an average density of 44 probes per Mb. This study demonstrates the ability to utilize existing cytogenomic platforms to guide the development of similar tools for species for which robust sequence assembly resources are not yet available. In the absence of a comprehensive, anchored and annotated feline genome sequence assembly at the outset of this study, we used an existing dog array with ~13kb probe spacing to aid selection of appropriate feline sequences for the design of oligonucleotide probes along the length of each cat chromosome. By its nature, this approach resulted in a reduction in relative effective resolution due to incomplete representation of all chromosomal regions within the evolving cat reference assembly, and the consequent absence of sequence data for direct orthologs of a subset of canine probe sequences. Despite these confounding factors, the resulting feline array design yielded a mean interval of 22.6 kb between consecutive probes, representing a ~350-fold increase in resolution compared to our previous feline CGH platform [7]. This resource provides a rapid means for screening feline tumors for somatic DNA copy number imbalances, for determining their impact on gene dosage, and in turn for elucidating the underlying molecular pathogenesis of the disease. While recent developments now permit the direct identification of copy number variations from next-generation sequencing (NGS) analysis of constitutional DNA, this approach acquires considerable analytical complexity when applied to cancer genomes. Confounding factors include the need for accurate normalization to account for the relative abundance of non-balanced genomic regions in tumor specimens, DNA degradation and fixation artefacts associated with archival specimens, and the presence of structural chromosomal rearrangements, polyploidy, polyclonality and ‘contaminating’ non-tumor DNA. Each of these factors contribute to the challenge of robust read alignment to the reference assembly for tumor specimens, and of sensitive and accurate detection of tumor-associated CNAs from NGS data. By its nature, the kinetics of the CGH technique means that these factors have a more moderate impact on the detection of copy number imbalances. Consequently, CGH continues to act as the ‘gold standard’ for the diagnosis of several human genomic abnormalities, both constitutional and somatic, and for identification of clinically actionable molecular targets [17]. 

Using our feline microarray design, competitive hybridization of constitutional DNA from clinically healthy male and female cats demonstrated the expected pattern of autosomal balance. In turn, copy number assessment along fcaX detected relative imbalances that were highly concordant with theoretical values for female versus male DNA comparisons. The sex-mismatch analysis also enabled delineation of the feline PAR boundary to a 25.7 kb interval between the *SHROOM2* and *WWC3* genes, approximately 7.3 Mb from fcaXptel. This concurs with the findings of Murphy et al. [18], who used radiation hybrid mapping to refine the boundary to a < 200kb region between these two genes. Using a combination of FISH and in-silico mapping analyses, Young et al. [19] mapped the canine PAR boundary to a 2 kb interval within the *SHROOM2* gene, approximately 6.6 Mb from cfaXptel. The observations from oaCGH analysis of fcaX from the present study are therefore fully concordant with prior comparative studies [20], supporting the markedly increased size of the feline PAR relative to that of human (~2.7 Mb), and the highly conserved localization of the canine and feline PAR boundary.

Two unexpected regions of DNA copy number imbalance were identified in normal sex-mismatch hybridizations. One, located on fcaA3ptel, indicated a discrete, high amplitude copy number loss of the *TETY1* locus in the female reference relative to the male. An earlier study [21] determined that the *TETY1* locus is encoded in multiple copies on fcaYq. Interestingly, however, a region of degenerate sequence homology with *TETY1* was found on fcaA3, suggestive of an ancestral autosomal origin for the Y-borne locus [21]. Furthermore, cross-species alignment of the *TETY1* sequence identified orthology with autosomal sequences from cfa24q25, consistent with known regions of conserved sequence between this dog chromosome region and fcaA3 [3,4]. The absence of orthologous sequences on the human or domestic dog Y chromosomes was taken to suggest that the transposition of *TETY1* from autosome to Y chromosome occurred during carnivore evolution after the divergence of ancestral cat and dog lineages [21]. The presence of multiple copies of *TETY1* on fcaY, the absence of Y-specific probes on feline and canine oaCGH platforms (due to the female origins of their reference genome sequence assemblies), and the existence of a highly conserved sequence on fcaA3, therefore explains the apparent copy number loss of this latter region in females versus males in sex-mismatch oaCGH analysis. A second region of relative copy number decrease on fcaF2 in the female reference showed partial overlap with the *RUNX1T1* gene; however at present there is no obvious explanation for this observation, and it is possible that this merely represents a reference genome assembly artefact.

The performance of the oaCGH array was evaluated further through analysis of an aggressive injection-site sarcoma, ISS-19a, which was examined at low resolution in an earlier study [7]. oaCGH analysis identified a broad distribution of genomic imbalance, including a complex profile of interrupted segmental copy number aberrations along fcaD1, with a peak of high-level amplification evident at fcaD1ptel. This pattern shares similarities with profiles associated with chromothripsis/chromoanasynthesis observed in some human cancers. These complex forms of intrachromosomal reorganization comprise alternating segmental copy number imbalances interspersed between regions of euploidy, and are thought to manifest simultaneously from highly localized, catastrophic genomic disruption events [22,23]. While the increase in probe density further delineates the discrete inflection points of these complex segmental changes, the large physical size of the high-level copy number gain on fcaD1ptel challenges assessment of the potential pathogenic significance of copy number amplification. Pertinent candidate targets of this ~11 Mb amplification include the platelet-derived growth factor D (*PDGFD*) gene on fcaD1:2.37 Mb and members of the matrix metalloproteinase (*MMP*) gene cluster on fcaD1:1.36 Mb, due to their varied roles in regulation of inflammation, wound healing, cell proliferation, migration, invasion, differentiation, angiogenesis and apoptosis [24]. Of note, multiple *MMP* genes have been shown to be upregulated in feline ISS, supporting an underlying role for inflammation in tumor pathogenesis [25]. Given the intimate association between increased gene dosage and transcriptional/translational upregulation in human cancer studies [26], it will be interesting to establish whether this complex reorganization of fcaD1 is conserved in other ISS cases. Combined molecular analysis showed that ISS-19a also presented with *KIT* amplification, apparent homozygous deletion of *PTEN*, *RB1* and *FAS*, and grossly balanced *CDKN2A/B* copy number. These same alterations were also identified in ISS-19b, a second mass obtained from the same patient 11 months after surgical resection of the first tumor. Evaluation of additional cases will be required to determine whether these represent potentially clinically relevant aberrations that may drive ISS pathogenesis, or whether they represent the accumulation of passenger alterations. Regardless, the close conservation in the genome-wide oaCGH profiles of ISS-19a and ISS-19b provide strong support for a common origin. 

Prior low-resolution CGH analysis also detected complex amplification and structural reorganization involving fcaE1ptel in ISS-19a [7]. In the present study, oaCGH determined that this amplification extends 13.2 Mb from the fcaE1p telomere of both ISS-19a and ISS-19b. Additionally, FISH analysis with a probe representing fcaE1ptel identified multiple hybridization sites resembling satellite-like sequences on 3–5 derivative chromosomes in each cell scored (Figure 5). It has been proposed that amplification of satellite DNA may be responsible for the induction of structural and numerical chromosome instability in feline fibrosarcoma, through aberrant kinetochore formation [27]. The detection of a similar high-level amplification in additional cases would provide further support for its potential biological significance. A small number of additional studies have used conventional chromosome banding techniques to examine feline fibrosarcomas. These have revealed complex karyotypic instability comprising a wide variety of structural abnormalities, and aberrant chromosome numbers ranging from mild hypodiploidy to marked hyperdiploidy (summarized in [28]). In combination, these findings show that feline fibrosarcomas exhibit a propensity towards major karyotypic disruption, which is a hallmark feature of many human soft tissue sarcomas, especially those associated with an aggressive phenotype [29].

The resource described here offers a series of key advantages over our first-generation microarray platform [7], most notably the ~350-fold increase in resolution. The increased probe density detected discrete relative DNA copy number imbalances in female versus male reference DNA hybridizations. Their identification will allow these regions to be characterized as natural polymorphisms in future studies, distinct from somatic alterations of tumor specimens. The in-silico design approach, using existing canine microarray probes to develop feline orthologs, can be exploited for direct comparison of evolutionarily-conserved genomic regions in both species. This, in conjunction with the increased resolution of our new microarray, provided accurate delineation of the feline PAR boundary, and revealed close conservation with that of the dog. These factors also allowed us to ‘drill down’ into large tumor-associated CNAs to reveal compelling gene targets within these intervals, such as the high-level amplification of fcaD1ptel in ISS-19. The increase in effective resolution also revealed the remarkably conserved profiles of complex, interrupted genomic gain and loss along fcaD1 in ISS-19a and ISS-19b, supporting the origin of the latter as a regrowth of the former, primary lesion. Interestingly, however, we also identified subtle differences between these profiles that demonstrate the potential for using this microarray platform to study tumor progression. Furthermore, our new microarray utilizes oligonucleotide probes that offer superior sensitivity and specificity for CNA detection compared to the large insert genomic clones used in our first-generation platform [17]. The use of oligonucleotide probe technology will also enable rapid and cost-effective production of custom iterations of the array design, to address the specific goals of future studies.

## 5. Conclusions

The chaotic nature of the genomic profiles observed in the present study reinforces the immense challenge of identifying and characterizing recurrent chromosome aberrations in complex karyotypes. The application of the feline oaCGH array to large cohorts of tumor cases should therefore offer a substantial advancement in recognition of non-random genomic events that underlie tumor pathogenesis. Future goals will include further development of the microarray platform, using the comprehensive felCat9 genome assembly to increase the density of probes in intervals that are currently underrepresented due to the incomplete status of earlier assemblies. It will also permit an increase in probe density for regions of specific biological interest, such as key cancer-associated genes, or highly recurrent CNAs. The consequent increase in resolution will aid identification of key drivers of disease pathogenesis, especially for copy number imbalances spanning large intervals. The availability of assembled sequence data from the feline Y chromosome [30] now also offers the opportunity to include male-specific content on the microarray. This will permit identification of somatic alterations involving this chromosome, and also provide a means to identify constitutional defects of sex chromosome complements. Ultimately, the identification of both generalized and subtype-associated genomic markers may aid the development of novel techniques for tumor diagnosis that guide the prognosis and selection of the optimal therapeutic approach for each patient. In turn, the identification of specific genes whose dosage and/or function is disrupted by chromosomal aberrations may clarify the precise genetic pathways involved in tumor development and progression, provide targets for novel therapeutic approaches, and enhance the integration of the domestic cat into advances in molecular diagnostics.

## Figures and Tables

**Table 1:** Summary of probe distribution on the feline oaCGH array. The physical size of each chromosome is shown along with the corresponding number of validated probes developed for the final array design. The mean and median probe spacing demonstrates the global comparability of probe distribution for each chromosome, with an overall 22.6kb spacing at the genome-wide level, equating to an average of 44 probes per Mb of sequence. The lower values for maximum spacing on fcaF1 and F2 reflect the acrocentric nature of these chromosomes, while the remaining chromosomes, all of which are biarmed, are impacted more heavily by the confounding effect of centromeric repeat sequence on probe design.

Chromosome	Chromosome Size (bp)	Total Number of Probes	Mean Spacing (bp)	Median Spacing (bp)	Maximum Spacing (bp)	Probes/Mb
A1	242,100,913	10,648	22,664	17,125	2,635,625	44
A2	171,471,747	7944	21,469	15,552	2,272,323	46
A3	143,202,405	6653	21,434	16,188	2,630,398	46
B1	208,212,889	9420	22,001	16,824	2,362,299	45
B2	155,302,638	7160	21,603	16,296	2,303,124	46
B3	149,751,809	7026	21,173	15,704	2,516,731	47
B4	144,528,695	6559	21,942	16,343	2,103,152	45
C1	222,790,142	10,724	20,646	15,770	2,108,648	48
C2	161,193,150	7462	21,511	16,288	3,159,135	46
D1	117,648,028	5174	22,610	16,463	2,184,516	44
D2	90,186,660	3863	23,132	16,397	2,155,220	43
D3	96,884,206	4275	22,564	16,227	2,223,652	44
D4	96,521,652	4249	22,622	16,361	2,213,074	44
E1	63,494,689	2712	22,955	15,407	2,301,848	43
E2	64,340,295	2352	27,026	18,082	2,536,363	37
E3	44,648,284	1561	28,241	17,413	3,394,005	35
F1	71,664,243	3082	22,498	16,523	650,963	43
F2	85,752,456	4137	20,102	15,601	333,037	48
X	130,557,009	5455	23,800	15,598	2,487,257	42
Total	2,460,251,910	110,456	22,631	-	-	44 (mean)

## References

[1] Murphy W.J. (2006). The feline genome. Genome. Dyn..

[2] Online Mendelian Inheritance in Animals. https://omia.org/.

[3] Lindblad-Toh K., Wade C.M., Mikkelsen T.S., Karlsson E.K., Jaffe D.B., Kamal M., Clamp M., Chang J.L., Kulbokas E.J., Zody M.C. (2005). Genome sequence, comparative analysis and haplotype structure of the domestic dog. Nature.

[4] Pontius J.U., Mullikin J.C., Smith D.R., Lindblad-Toh K., Gnerre S., Clamp M., Chang J., Stephens R., Neelam B., Volfovsky N. (2007). Initial sequence and comparative analysis of the cat genome. Genome. Res..

[5] LeBlanc A.K., Breen M., Choyke P., Dewhirst M., Fan T.M., Gustafson D.L., Helman L.J., Kastan M.B., Knapp D.W., Levin W.J. (2016). Perspectives from man’s best friend: National Academy of Medicine’s Workshop on Comparative Oncology. Sci. Transl. Med..

[6] Schiffman J.D., Breen M. (2015). Comparative oncology: What dogs and other species can teach us about humans with cancer. Philos. Trans. R. Soc. Lond. B. Biol. Sci..

[7] Thomas R., Valli V.E., Ellis P., Bell J., Karlsson E.K., Cullen J., Lindblad-Toh K., Langford C.F., Breen M. (2009). Microarray-based cytogenetic profiling reveals recurrent and subtype-associated genomic copy number aberrations in feline sarcomas. Chromosome. Res..

[8] Buckley R.M., Davis B.W., Brashear W.A., Farias F.H.G., Kuroki K., Graves T., Hillier L.W., Kremitzki M., Li G., Middleton R. (2020). A new domestic cat genome assembly based on long sequence reads empowers feline genomic medicine and identifies a novel gene for dwarfism. BioRxiv.

[9] Poorman K., Borst L., Moroff S., Roy S., Labelle P., Motsinger-Reif A., Breen M. (2015). Comparative cytogenetic characterization of primary canine melanocytic lesions using array CGH and fluorescence in situ hybridization. Chromosome. Res..

[10] Shapiro S.G., Raghunath S., Williams C., Motsinger-Reif A.A., Cullen J.M., Liu T., Albertson D., Ruvolo M., Bergstrom Lucas A., Jin J. (2015). Canine urothelial carcinoma: Genomically aberrant and comparatively relevant. Chromosome. Res..

[11] Thomas R., Borst L., Rotroff D., Motsinger-Reif A., Lindblad-Toh K., Modiano J.F., Breen M. (2014). Genomic profiling reveals extensive heterogeneity in somatic DNA copy number aberrations of canine hemangiosarcoma. Chromosome. Res..

[12] Thomas R., Demeter Z., Kennedy K.A., Borst L., Singh K., Valli V.E., Le Boedec K., Breen M. (2017). Integrated immunohistochemical and DNA copy number profiling analysis provides insight into the molecular pathogenesis of canine follicular lymphoma. Vet. Comp. Oncol..

[13] Kent W.J. (2002). BLAT--the BLAST-like alignment tool. Genome. Res..

[14] Zhang Z., Schwartz S., Wagner L., Miller W. (2000). A greedy algorithm for aligning DNA sequences. J. Comput. Biol..

[15] Larkin M.A., Blackshields G., Brown N.P., Chenna R., McGettigan P.A., McWilliam H., Valentin F., Wallace I.M., Wilm A., Lopez R. (2007). Clustal W and Clustal X version 2.0. Bioinformatics.

[16] Thomas R., Seiser E.L., Motsinger-Reif A., Borst L., Valli V.E., Kelley K., Suter S.E., Argyle D., Burgess K., Bell J. (2011). Refining tumor-associated aneuploidy through ’genomic recoding’ of recurrent DNA copy number aberrations in 150 canine non-Hodgkin lymphomas. Leuk. Lymphoma..

[17] Cheung S.W., Bi W. (2018). Novel applications of array comparative genomic hybridization in molecular diagnostics. Expert. Rev. Mol. Diagn..

[18] Murphy W.J., Davis B., David V.A., Agarwala R., Schaffer A.A., Pearks Wilkerson A.J., Neelam B., O’Brien S.J., Menotti-Raymond M. (2007). A 1.5-Mb-resolution radiation hybrid map of the cat genome and comparative analysis with the canine and human genomes. Genomics.

[19] Young A.C., Kirkness E.F., Breen M. (2008). Tackling the characterization of canine chromosomal breakpoints with an integrated in-situ/in-silico approach: The canine PAR and PAB. Chromosome. Res..

[20] Raudsepp T., Chowdhary B.P. (2015). The Eutherian Pseudoautosomal Region. Cytogenet. Genome. Res..

[21] Murphy W.J., Pearks Wilkerson A.J., Raudsepp T., Agarwala R., Schaffer A.A., Stanyon R., Chowdhary B.P. (2006). Novel gene acquisition on carnivore Y chromosomes. PLoS. Genet..

[22] Zhang C.Z., Leibowitz M.L., Pellman D. (2013). Chromothripsis and beyond: Rapid genome evolution from complex chromosomal rearrangements. Genes. Dev..

[23] Storchova Z., Kloosterman W.P. (2016). The genomic characteristics and cellular origin of chromothripsis. Curr. Opin. Cell. Biol..

[24] Cathcart J., Pulkoski-Gross A., Cao J. (2015). Targeting Matrix Metalloproteinases in Cancer: Bringing New Life to Old Ideas. Genes. Dis..

[25] Sorensen K.C., Kitchell B.E., Schaeffer D.J., Mardis P.E. (2004). Expression of matrix metalloproteinases in feline vaccine site-associated sarcomas. Am. J. Vet. Res..

[26] Durrbaum M., Storchova Z. (2016). Effects of aneuploidy on gene expression: Implications for cancer. FEBS. J..

[27] Santos S., Chaves R., Adega F., Bastos E., Guedes-Pinto H. (2006). Amplification of the major satellite DNA family (FA-SAT) in a cat fibrosarcoma might be related to chromosomal instability. J. Hered..

[28] von Erichsen J., Hecht W., Lohberg-Gruene C., Reinacher M. (2012). Cell lines derived from feline fibrosarcoma display unstable chromosomal aneuploidy and additionally centrosome number aberrations. Vet. Pathol..

[29] Dufresne A., Cassier P., Heudel P., Pissaloux D., Wang Q., Blay J.Y., Ray-Coquard I. (2015). Molecular biology of sarcoma and therapeutic choices. Bull. Cancer..

[30] Brashear W.A., Raudsepp T., Murphy W.J. (2018). Evolutionary conservation of Y Chromosome ampliconic gene families despite extensive structural variation. Genome. Res..

